# Relationship between antipsychotic medication and aggressive events in patients with a psychotic disorder hospitalized for treatment

**DOI:** 10.1186/s12888-023-04692-1

**Published:** 2023-03-28

**Authors:** Georgia Tseligkaridou, Stephan T. Egger, Tobias R. Spiller, Lena Schneller, Fritz Frauenfelder, Stefan Vetter, Erich Seifritz, Achim Burrer

**Affiliations:** 1 Department of Psychiatry, Psychotherapy and Psychosomatics, Psychiatric Hospital, University of Zurich, Zurich, Switzerland; 2 Legal and Compliance, Psychiatric Hospital, University of Zurich, Zurich, Switzerland; 3 Department of Nursing, Therapies and Social Work, Psychiatric Hospital, University of Zurich, Zurich, Switzerland

**Keywords:** Schizophrenia, psychosis, aggression, Antipsychotic, Dopamine affinity, SOAS-R

## Abstract

**Background:**

Disruptive and aggressive behavior is frequent in patients with a psychotic disorder; furthermore, it is a recurrent reason for compulsory admission. Even during treatment, many patients continue to show aggressive behavior. Antipsychotic medication is posed to have anti-aggressive properties; its prescription is a common strategy for the treatment (and prevention) of violent behavior. The present study aims to investigate the relation between the antipsychotic class, according to the dopamine D2-Receptor binding affinity (i.e., “loose” – “tight binding”), and aggressive events perpetrated by hospitalized patients with a psychotic disorder.

**Methods:**

We conducted a four-year retrospective analysis of legally liable aggressive incidents perpetrated by patients during hospitalization. We extracted patients’ basic demographic and clinical data from electronic health records. We used the Staff Observation Aggression Scale (SOAS-R) to grade the severity of an event. Differences between patients with a “loose” or “tight-binding” antipsychotic were analyzed.

**Results:**

In the observation period, there were 17,901 direct admissions; and 61 severe aggressive events (an incidence of 0.85 for every 1,000 admissions year). Patients with a psychotic disorder perpetrated 51 events (incidence of 2.90 for every 1,000 admission year), with an OR of 15.85 (CI: 8.04–31.25) compared to non-psychotic patients. We could identify 46 events conducted by patients with a psychotic disorder under medication. The mean SOAS-R total score was 17.02 (2.74). The majority of victims in the “loose-binding” group were staff members (73.1%, n = 19), while the majority of victims in the “tight-binding” group were fellow patients (65.0%, n = 13); (*X*^2^(3,46) = 19.687; p < 0.001). There were no demographic or clinical differences between the groups and no differences regarding dose equivalents or other prescribed medication.

**Conclusions:**

In aggressive behaviors conducted by patients with a psychotic disorder under antipsychotic medication, the dopamine D2-Receptor affinity seems to have a high impact on the target of aggression. However, more studies are needed to investigate the anti-aggressive effects of individual antipsychotic agents.

## Background

Although violence and aggression are not easy to define and delimit; they are understood as the intentional use of physical force, verbal or non-verbal abuse or threats against another person that either result in (or has a high likelihood of resulting) injury, death, or psychological harm [1, 2]. The origins and functions of violence and aggression are the subjects of century-long research and debate. A certain proportion of patients with psychotic disorders exhibits aggressive or violent behavior. Among patients with a psychotic disorder violence is highly associated with particular demographic and premorbid risk factors such as history of being victimized, male gender, homelessness, history of assault or arrest and psychopathological factors like poor impulse control and lack of insight, comorbid substance misuse and non-adherence[3]. The fact that aggressive behavior occurs in a minority of patients with a psychotic disorder is also contributing to stigmatization of psychiatric patients and treatment by generalizing the connection between violence and mental illness.

In mental disorders, violence and aggression are common in patients with a psychotic disorder [4, 5]. Furthermore, disruptive and aggressive behavior is a frequent reason for compulsory admissions to psychiatric treatment. Even during hospitalization, a significant proportion of patients continues to show aggressive behavior [6]. A large majority of staff from psychiatric wards have experienced aggression and violence from patients, principally verbal and physical assaults, but also sexual harassment [7]. Also, other patients and relatives are not seldom victims of violence.

Violence and aggression in psychiatric wards pose a great challenge for treatment and a juristic liability for service providers. Moreover, aggressive events during hospitalization are detrimental for everyone involved. Not only because they pose a risk for injury (and even traumatization) for the victims but also because they have adverse effects on the perpetrators, including the use of force for involuntary medication, isolation or restraint [8]. Therefore, the identification and prevention of risk factors for aggression is crucial during psychiatric hospitalization. In this context, aggression management and contention programs were continuously developed and implemented. These programs frequently comprise the standardized monitoring of disruptive and aggressive behavior [9], early intervention methods such as “talking down,” and the proper use of containing and force in the case of an aggressive event [10].

It is claimed that psychiatric medication, particularly individual antipsychotic agents, have an anti-aggressive effect [11–13]. For clozapine a particular anti-aggressive effect is reported in several studies [14, 15]. Therefore, antipsychotics are frequently prescribed in patients with aggressive behavior. However, the extent to which all antipsychotics can produce this effect and any differences among them remain unclear. Despite this, it is widely acknowledged that the blockade of the dopamine D2 receptor plays a crucial role in the efficacy of antipsychotics, as evidenced by their mechanism of action. Nevertheless, it is important to note that the dopamine binding profile of modern antipsychotics varies among the different substances, highlighting the need for further investigation [16]. Moreover, it has been noted that the subjective and nuanced effects of antipsychotics are perceived differently by both patients and healthcare professionals. However, the impact of these differences on the manifestation of violent and aggressive behavior remains uncertain, to the best of our knowledge.

This study aimed to analyze the relationship between antipsychotic medication and aggressive behavior in (medicated) patients with a psychotic disorder using a retrospective, explorative design. In particular, we analyzed the relationship between the type of pre-existing antipsychotic medication and the characteristics of the aggressive event. Unfortunately, inconsistent definitions and delimitation of violent and aggressive behavior make comparison and generalization difficult [17]. Therefore, we defined a violent and aggressive event according to its medico-legal implication and judicial liability.

## Methods

### Study design and sample

The current study is a retrospective analysis of the characteristics of aggressive events perpetrated by adult (i.e., 18–65 years) patients with a psychotic disorder during their hospitalization in a psychiatric ward of the Department of Psychiatry, Psychotherapy and Psychosomatics of the University Hospital of Psychiatry Zurich. For all patients admitted between January 2018 and December 2021, we reviewed the clinical incident system and extracted their demographic and clinical data from the electronic medical record.

The study was conducted in accordance with the Declaration of Helsinki, and approved by the Ethics Committee of the Canton of Zurich (BASEC-Nr. 2021 − 01246). Patient consent was waived due to approval by the Ethics Committee of the Canton of Zurich.

The University Hospital of Psychiatry Zurich is a public hospital with a service mandate for psychiatric care covering a mixed urban and rural region of approximately 500,000 inhabitants. It offers in- and outpatient treatment for adults with a psychiatric disorder. Since the definition and operationalization of violence and aggression in the medical field are heterogeneous, we decided to use the definition as it is anchored in Swiss legislation (illegal actions with damage to objects or threats or physical injuries to other people). Furthermore, due to legal and liability requirements, all such events are monitored and systematically reported to a clinical incident system.

### Demographics, diagnoses, and measurement instruments

For analysis, we included basic demographic data (i.e., age, sex, highest education, and housing condition). Diagnoses were made using the WHO ICD-10 diagnostic criteria; we included the Health of the Nation Outcome Scale (HoNOS); and the Staff Observation Aggression Scale (SOAS-R) in its revised version.

The main diagnosis was made during hospitalization according to the ICD-10 diagnostic criteria by the treating physician or psychologist and confirmed by a board-certified psychiatrist. For the analysis, we included all diagnoses with psychotic features since these diagnoses exhibit a clear indication for the prescription of an antipsychotic drug. Therefore, diagnoses of interest included: schizophrenia (F20); delusional disorder (F22); brief psychotic disorder (F23); schizoaffective disorder (F25); mania with psychotic features (F30.2); and bipolar disorder with psychotic features, either manic (F31.2) or depressive (F31.5) episode. Existing comorbid diagnoses of substance use disorder were also made according to the ICD-10 criteria. (i.e. intoxication, harmful use, dependence or withdrawal). For the analysis the following substances were included alcohol use disorder (ICD-10: F10); opioid use disorder (ICD-10: F11); cannabis use disorder (ICD-10: F12); benzodiazepine use disorder (ICD-10: F13); cocaine use disorder (ICD-10: F14); and stimulant use disorder (ICD-10: F15).

The Health of the Nation Outcome Scales (HoNOS) is a measurement instrument used to assess the severity and treatment requirements of patients with a psychiatric disorder; it has become a widely used evaluation tool, and in some countries, it is a mandatory outcome measure. The HoNOS evaluates 12 domains covering behavior, symptomatology, impairment, and psychosocial functioning. Each item is rated on a five-point Likert scale from 0 (“no problem”) to 4 (“severe to very severe problem”). We evaluated the HoNOS at scale level (i.e., sum score ranging from 0 to 48) [18–21]. Furthermore, we considered HoNOS items rated as three or four clinically significant and integral to the patient’s care plan [21].

The Staff Observation Aggression Scale-Revised Version (SOAS-R) was developed to measure the nature and severity of aggressive events [22]; it has become a widely used and validated tool for measuring inpatient aggression [22, 23]. A detailed scoring system allows characterize and quantify the severity of an aggressive event [24]. Therefore, events are rated in five domains, with several categories, each assigned a different grading. The maximum score in each domain is chosen, and a sum score builds- the SOAS-R ranges from 0 to 22 points. The severity of the event can be categorized as mild (0–7 points), moderate (8–15 points), and severe (12–22 points) [25].

The first domain of the SOAS- R appraises whether there was a provocation leading to the aggressive event, it is scored from 0 to 2 points. A score of zero (“0”) indicates that the event was provoked through waiting, receiving help in daily activities, or the patients being denied something or provoked by other patients. A score of one (“1”) is given if there is no obvious or understandable provocation; a score of two (“2”) is given if the event is provoked through the request or instruction of the staff (e.g., requiring the patient to take medication).

The second domain rates the means used by the patient and can be scored from 0 to 3 points. A score of zero (“0”) indicates verbal aggression or threats. A score of one (“1”) is given if aggressive events involve the use of ordinary non-dangerous objects (e.g., furniture). A score of two (“2”) is given if there is bodily aggression (e.g., use of punch or kick). Finally, a score of three (“3”) is given if weapons, dangerous objects, or methods are used (e.g., knife, strangulation).

The third domain rates the target of aggression scored from 0 to 4 points. A score of zero (“0”) is given if there is no target of aggression. A score of one (“1”) is given if the target of aggression is an object (e.g., furniture). If the aggression involves other patients, a score of two (“2”) is given. A score of three (“3”) is given if the target of aggression are staff members (i.e., nurses, doctors, cleaning or security staff). Finally, a score of four is given if the targets of aggression are visitors or strangers to the ward (e.g., relatives, police).

The fourth domain rates the consequences for the victim(s); it is scored from 3 to 9 at three-point intervals (i.e., “0;” “3;” “6;” or “9”). Zero (“0) represents no harm or damage. Three (“3”), the object was damaged. Six (“6”) of the affected person(s) felt threatened. Finally, nine (“9”), there was an injury or pain requiring treatment.

The fifth and last domain rates the measures used to stop aggression, with a score between 0 and 4 in two-point intervals (i.e., “0;“ “2;“ or “4”). A score of zero (“0”) is given if talk down is sufficient and no other measures are necessary. A score of two (“2”) points is given if medication (peroral or parenteral) is given; or if the police have to be involved. Finally, a score of four (“4”) is given if the use of force is necessary for seclusion/isolation or restraint.

### Medication

Antipsychotic medication was characterized according to their action on the dopamine D2 receptor affinity into two categories (see Table 1): Loose binding (e.g., olanzapine); or tight binding (e.g., risperidone) according to their dissociation constant Ki for the dopamine D2 receptor. If Ki is higher than the one of dopamine itself, the antipsychotic medication is classified as tight binding; and as loose binding, if their dissociation constant is lower than the one of dopamine itself [16, 26, 27].

We assessed whether the patients were treated with an antipsychotic monotherapy or a combination of antipsychotics; or additionally also received an anxiolytic (i.e., benzodiazepines) or mood stabilizers (e.g., lithium, valproate). We calculated the Daily Defined Dose for each prescribed psychopharmacological agent and the Chlorpromazine Equivalents for the prescribed antipsychotic drugs. Only medication continuously taken in the past three days was considered. In case of changing doses, the higher dose was chosen. If patients took a combined antipsychotic therapy of a loose binding and tight binding antipsychotic they were grouped into the group of the agent with the higher CPZ-equivalent dose.

**Table 1:** Characteristics of antipsychotics prescribed to the sample population compared to dopamine

	Ki value nM Dopamine D2 receptor	Daily Defined Dose (DDD)	Chlorpromazine Equivalent Ratio (EQ)
**Loose binding or partial agonism**			
Quetiapine^a^	122	400 mg	0.80
Clozapine^a^	63	300 mg	1.50
Cariprazine^c^	18.0	3 mg	100
Olanzapine^a^	5.1	10 mg	30
Amisulpride^b^	1.8	400 mg	0.86
Aripiprazole^b^	1.8	15 mg	20.0
**Tight binding**			
Dopamine^a^	1.75	-	-
Lurasidone^c^	1.68	60 mg	16
Paliperidone^b^	1.6	6 mg/2.5 mg	66.7
Risperidone^a^	1.1	5 mg/1.8 mg	100
Haloperidol^a^	0.55	8 mg	60.0
Zuclopenthixol^d^	Comparable to Haloperidol	30 mg/15 mg	12

^a^Ki values derived from: [16]

^b^Ki values for derived from: [28]

^c^Ki values derived from the public PDSP Database

^d^[27]

### Statistical analysis

The demographic and clinical characteristics of the sample were analyzed using descriptive statistics; results are presented with mean (standard deviation) and proportions. In the first step, the sample was classified according to the type of prescribed antipsychotic drug in “loose binding” or “thigh binding.” Differences between both groups were analyzed: we used non-parametric testing (due to the small sample size) for continuous variables, with the Mann-Whitney U for independent samples. For differences in proportions, Chi-Square tests were used. For positive results, a subsequent Chi-Square omnibus comparison for the different subcategories was performed if pertinent. We calculated the correlation between the antipsychotic groups and the characteristics of the aggressive events. For differences found between the groups, we calculated their probabilities and their relationship with demographic and clinical variables using the general linear model. All tests were performed two-sided, with an alpha level of p < 0.05. Since the study was designed exploratively, adjustments for multiple comparisons were not made. The analysis was performed in the R environment (version 4.2.1).

## Results

### Sample characteristics

In the observation period, there were 17,901 direct admissions; of those, 4,394 had a diagnosis of interest for the analysis, and there were 61 critical incidents involving aggressive behavior by patients, leaving an incidence of 3.4 events for every 1,000 admissions. Patients with a psychotic disorder perpetrated 51 of the aggressive events, resulting in an incidence of 2.90 for every 1,000 admissions year. Patients with a psychotic disorder (or a disorder with psychotic features) had an OR of 15.85 (CI: 8.04–31.25) for perpetrating a severe aggressive event compared to non-psychotic patients. For the final analysis, five patients with a psychotic disorder did not have any medication at the time of the event and were therefore excluded from further analysis. This resulted in a final sample size of 46 patients with a psychotic disorder that perpetrated a severe aggressive event.

The mean age of the patients included in the sample was 33.9 (SD 12.8) years; the majority were male (76.1%, n = 35). Over two-thirds (69.6%, n = 32) of patients had a compulsory admission order. Most patients had over ten previous hospitalizations, 11.9 (16.6) with a right skewed distribution (median of 6; IQR: 11). Diagnoses of alcohol and substance use were recorded, the most frequently consumed (abused) substances were: cannabis (23.9%, n = 11); cocaine (19.6%, n = 9); alcohol (13.0%, n = 6); and opioids (10.9%, n = 5); the consumption of benzodiazepines and stimulants were both below 10%. The HoNOS score at admission was 26.59 (6.93). For further details, see Table 2.

**Table 2:** Demographic and clinical characteristics of the sample. (n, %)

	Total Sample n = 61	Included n = 46	Excluded n = 15
Age	33.00 (12.84)	33.87 (12.78)	30.58 (13.47)
Sex			
Female	14 (23.0)	11 (23.9)	3 (20.0)
Male	47 (77.0)	35 (76.1)	12 (80.0)
Diagnosis			
Neuro-Cognitive Disorder	1 (1.6)	0 (0)	1 (6.7)
Alcohol Use Disorder	2 (3.3)	0 (0)	2 (13.3)
Anxiety Disorder	1 (1.6)	0 (0)	1 (6.7)
Bipolar Disorder	9 (14.7)	7 (15.2)	2 (13.3)
Depressive Disorder	4 (6.6)	0 (0)	4 (26.7)
Schizophrenia	42 (68.9)	39 (84.8)	3 (20.0)
Personality Disorder	2 (3.3)	0 (0)	2 (13.3)
Compulsive Admission	37 (60.7)	32 (69.6)	5 (33.3)
HoNOS (Admission)	25.75 (7.29)	26.59 (6.93)	22.00 (8.12)

### Medication

Two-thirds (67.4%, n = 31) of patients had an antipsychotic monotherapy. Another third (32.6% n = 15) had a combination of two antipsychotic medications. There were 66 prescribed different antipsychotic medications, either as monotherapy or in combination. The most prescribed medication as either monotherapy or combination was olanzapine (21.2%, n = 14), followed by risperidone (16.6%, n = 11), paliperidone (16.6%, n = 11); and quetiapine (13.6%, n = 9). The majority of patients with monotherapy had olanzapine (22.6%, n = 7), followed by risperidone (19.4%, n = 6) and paliperidone (16.1%, n = 5). The most prescribed Long Acting Injectable was Paliperidone (n = 8); with just one patient prescribed Risperidone. The Daily Defined Dose (DDD) of antipsychotics was 1.80 (1.05), with 654.72 (498.26) Chlorpromazine Equivalents. For further details, see Table 3.

In 19 (41.3%) of all patients, an augmentating mood stabilizer was prescribed. In half of the patients them (52.6% n = 10) the mood stabilizer was prescribed in addition to an antipsychotic monotherapy. The most prescribed medication for augmentation was valproate (84.2%, n = 16); lithium (15.8%, n = 3) was marginally prescribed, and other augmentation strategies were not recorded. Over half of patients (58.7%, n = 27) were prescribed a benzodiazepine. The most frequently prescribed benzodiazepine was lorazepam (50%, n = 14), followed by diazepam (28.6%, n = 8); alprazolam and oxazepam were prescribed in three (10.7%) patients each. The DDD of anxiolytics was 1.15 (2.19); the DDD of mood stabilizers was 0.46 (0.70). The total DDD of psychopharmacologic drugs was 3.42 (2.52).

### Aggressive events

The time elapsed from admission to the aggressive event was 21.7 (27.7) days. The majority (69.6%, n = 32) of events occurred during the daytime. The SOAS-R total score was 17.02 (2.74). For the majority (65.2%, n = 30) of patients, the aggressive behavior did not have an obvious provocation. The means of aggression were mostly physical aggression (60.9%, n = 28) or objects used as weapons (37.0%, n = 17). The majority of victims of the aggression were either staff (50.0%, n = 23) or other patients (30.4%, n = 14); most of the victims had injuries (73.9%, n = 34) or felt threatened (15.2%, n = 7). In almost all cases (91.3%, n = 42), force had to be used to stop aggression.

### Comparison of loose binding and tight binding antipsychotic

Of the 46 patients included in the analysis, 26 (56.5%) had a prescribed “loose binding” antipsychotic drug (i.e., Amisulpride, Aripiprazole, Cariprazine, Clozapine, Olanzapine or Quetiapine), and 20 (43.5%) had a “tight binding” antipsychotic drug (i.e., Haloperidol, Lurasidone, Paliperidone, Risperidone or Zuclopenthixol). There was no difference between the “loose binding” and “tight binding” groups regarding age (35.8 ± 12.6 vs. 31.3 ± 12.81, p = 0.23); sex (73.1% vs. 80.0% males, p = 0.84). There was also no difference in alcohol or substance consumption: Cannabis (19.2% vs. 30.0%, p = 0.62); Cocaine (11.5% vs. 30.0%, p = 0.23); Alcohol (11.0% vs. 11.0%, p = 1); and Opioids (11.0% 10.5%, p = 1.0). There were also no differences regarding compulsive admission (61.5% vs. 80.0%, p = 0.31), the number of past admissions (12.27 ± 20.15 vs. 9.55 ± 10.75, p = 0.59), the HoNOS at admission was similar (27.52 ± 7.20 vs. 25.37 ± 6.55, p = 0.31): without any difference at subscale or item level. There were also no differences in length of stay (59.08 ± 67.35 vs. 83.70 ± 71.26 days, p = 0.24); time to the aggression (19.65 ± 23.92 vs. 24.40 ± 32.56 days, p = 0.57).

There were also no differences regarding antipsychotic monotherapy between the “loose binding” and “tight binding” (65.4%, vs. 70.0%, p = 0.81); combination (24.6% vs. 30.5%, p = 0.98); augmentation (50.0% vs. 30.0%, p = 0.24). The most frequent combination was with an antipsychotic from the opposite group (23.1% vs. 25.0%, p = 0.91). There were no differences between the groups using long-acting injectables (either as monotherapy or combination) (p = 0.15). There were no differences regarding the agents used for augmentation (p = 0.10) or the rate of prescribed anxiolytics (p = 0.27). There were no differences regarding DDD of antipsychotic drugs (1.87 ± 1.11 vs. 1.71 ± 0.98, p = 0.59); Chlorpromazine Equivalents (656.04 ± 432.88 vs. 653.00 ± 584.32, p = 0.98). There was no difference for the DDD of anxiolytics (1.29 ± 2.78 vs. 0.98 ± 1.06, p = 0.64); or the DDD of mood stabilizers (0.49 ± 0.67 vs. 0.42 ± 0.75, p = 0.72). There was also no difference in the total DDD of psychopharmacologic drugs (3.66 ± 3.04 vs. 3.11 ± 1.65, p = 0.47). For further details, see Table 3.

### Relationship between antipsychotic medication and aggressive events

**Fig. 1:**
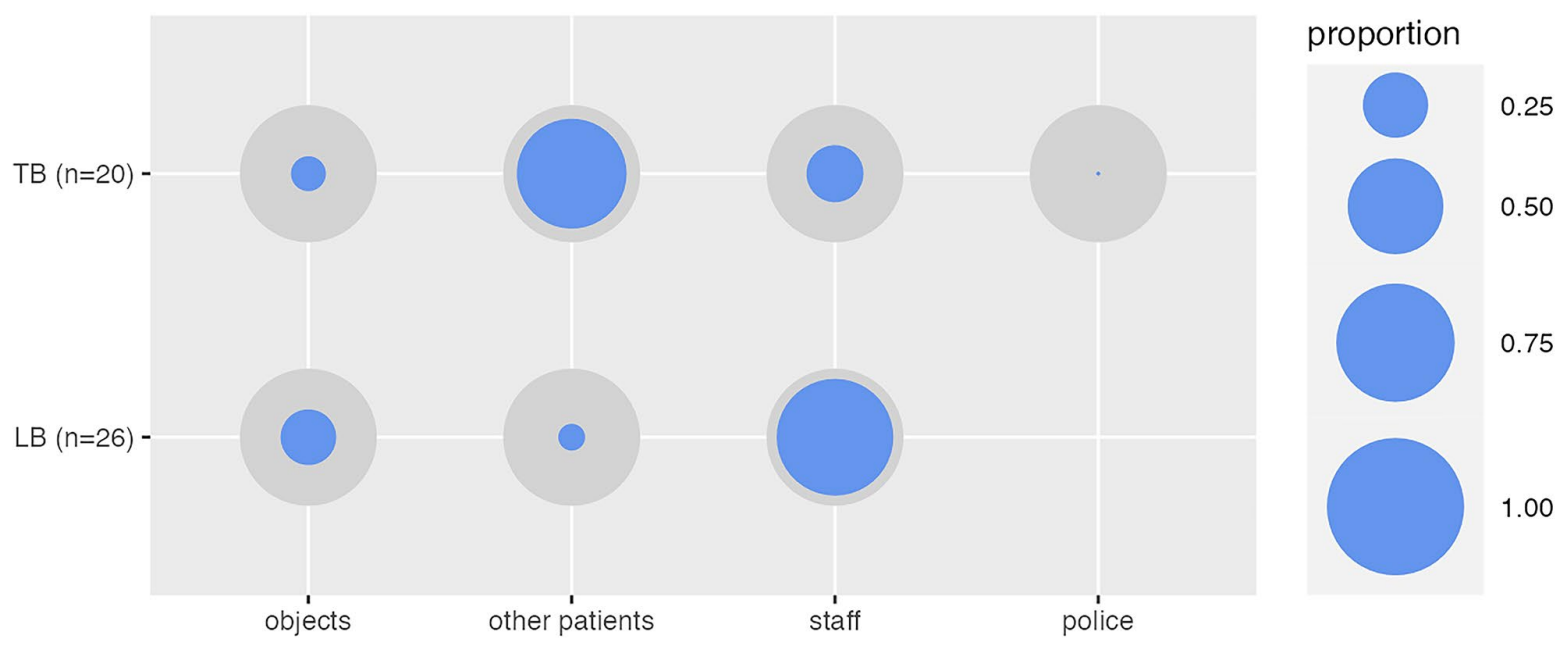
Proportion of the target of aggression (SOAS-R Item 3) according to the dopamine D2 Receptor affinity of the antipsychotic agent; TB: “Tight Binding”; LB: “Loose Binding.”

Regarding the SOAS-R, we could not find differences in total score (16.96 ± 3.14 vs. 17.10 ± 2.17, p = 0.87). There were also no differences regarding the provocation for the aggressive behavior (p = 0.31); the means of aggression were also identical (p = 0.39). The majority of victims of the aggression in the “loose binding” group were staff members (73.1%, n = 19), while in the “tight binding” agents, the most frequent victims of aggression were other patients (65.0%, n = 13). This difference reached statistical significance (*X*^2^ (3, 46) = 19.687; p < 0.001), and the results remained stable after Chi-Square omnibus testing for each category (Fig. 1). There were no differences regarding the consequences for the victims (p = 0.31) or the means used to stop aggression (p = 0.96). For further detail, see Table 4.

The Odds Ratio for an aggressive event against other patients with a “tight binding” antipsychotic drug was 22.3 (CI: 4.8–167.0). Inversely the risk for an aggressive event against a staff member for a “loose binding” antipsychotic drug was 10.86 (CI: 2.91–49.4). In a confirmatory analysis including the combination of “loose binding” and “tight binding” antipsychotics as an additional category, the relationship between the D2 binding antipsychotic and the target of aggression remained stable; however, for the case of the combination of both classes of antipsychotics the relation was inconclusive (i.e. statistically non-significant). However, in this approach the actual dose and more important the Chlorpromazine Equivalents of each antipsychotic medication were not considered.

Through the general linear model analysis, we could not find any demographic (age, sex), clinical (alcohol and substance use, HoNOS score), pharmacological (DDD of antipsychotics, DDD of mood stabilizers, DDD of anxiolytics, Chlorpromazine Equivalents of antipsychotics), or service use (compulsory admission, time of day, length of stay, time to event); triggers (SOAS-R Item-1); and characteristics of the aggressive event (remaining SOAS-R Items) variables modifying the relationship between the type of antipsychotic drug and the target of the aggression.

**Table 3:** Prescribed medications according to the D2 receptor affinity of the (main) prescribed antipsychotic: (n, %)

	Monotherapy		Combination		Augmentation Mood Stabilizer		Augmentation Anxiolytics	
	n = 31		n = 15		n = 19		n = 27	
**Tight Binding**								
Haloperidol	1 (3.2)		With Tight Binding		6 (31.6)		14 (51.9)	
Zuclopenthixol	2 (6.5)		3 (20.0)					
Risperidone	6 (19.4)		With Loose Binding					
Paliperidone	5 (16.2)		3 (20.0)					
Lurasidone	0 (0.0)							
**Loose Binding**								
Quetiapine	4 (12.9)		With Tight Binding		13 (68.4)		13 (48.1)	
Olanzapine	7 (22.6)		5 (33.3)					
Clozapine	1 (3.2)		With Loose Binding					
Amisulpride	2 (6.5)		4 (26.7)					
Aripiprazole	2 (6.5)							
Cariprazine	1 (3.2)							

**Table 4:** SOAS-R according to the type of prescribed antipsychotic drug. (n, %)

	Loose Binding	Tight Binding	
	n = 26	n = 20	
**SOAS-R 1 Provocation of Aggressive Behavior (n, %)**			p = 0.30
0 Daily Routine	9 (34.6)	5 (25.0)	
1 No obvious Provocation	15 (57.7)	15 (75.0)	
2 Disagreement	2 (7.7)	0 (0.0)	
**SOAS-R 2 Means Used by Aggressor (n, %)**			p = 0.39
0 Verbal Aggression/Threat	0 (0.0)	1 (5.0)	
2 Parts of the Body	15 (57.7)	13 (65.0)	
3 Dangerous Objects	11 (42.3)	6 (30.0)	
**SOAS-R 3 Target of Aggression (n, %)**			p < 0.001
0 None	0 (0.0)	0 (0.0)	
1 Furniture/Objects	5 (19.2)	2 (10.0)	
2 Other Patients	2 (7.7)	13 (65.0)	
3 Staff	19 (73.1)	4 (20.0)	
4 Police	0 (0.0)	1 (5.0)	
**SOAS-R 4 Consequences for victims (n, %)**			p = 0.30
0 None	0 (0.0)	0 (0.0)	
3 Object Damaged	4 (15.4)	1 (5.0)	
6 Felt Threatened	5 (19.2)	2 (10.0)	
9 Injury/Pain	17 (65.4)	17 (85.0)	
**SOAS-R 5 Measure to Stop Aggression (n, %)**			p = 0.96
0 None/Down Talk	1 (3.8)	1 (5.0)	
2 Medication	1 (3.8)	1 (5.0)	
4 Use of Force	24 (92.3)	18 (90.0)	
**SOAS-R Total (mean (SD))**	**16.96 (3.14)**	**17.10 (2.17)**	p = 0.87

## Discussion

The study was designed to analyze the relationship between the type of pre-existing antipsychotic medication and the characteristics of the aggressive event.

While it is known that there are anti-aggressive effects of antipsychotic medication in general and of clozapine in particular [13, 15], to the best of our knowledge, this is the first study to investigate the relationship between the type of antipsychotic medication and aggressive events in patients with a psychotic disorder using the Staff Observation Aggression Scale-Revised (SOAS-R) [24] as standardized event rating. This analysis aimed to assess the relationship between antipsychotic drugs, grouped by their affinity for the dopamine D2 receptor, and the characteristics of aggressive behavior in patients with a psychotic thought disorder. We found a strong relationship between the type of antipsychotic prescribed and the characteristics of aggression. In patients with a “tight binding” antipsychotic, the victim was more frequently another patient (65.0%, n = 13), while for “loose binding” antipsychotics, the victim was more frequently a staff member (73.1%, n = 19); p < 0.001.

The numbers of severe aggressive events during hospitalization are generally low; in the observation period, there were a total of 61 events (in 17.901 direct admissions); however, over 80% of the events are accountable to patients with a psychotic disorder; within these the majority with schizophrenia, and a few with a bipolar disorder. Most events were perpetrated through patients with a psychotic disorder, in the majority despite antipsychotic medication being initiated.

The severity of recorded events (mean SOAS-R of 17.02 (2.74)) was higher in our sample of psychotic patients than in a general sample from adult psychiatry in Germany reporting a mean SOAS-R of 11.79 (1.08) in patients with schizophrenia spectrum disorder [29].

Several factors contribute to aggression development and escalation, reflecting the difficulty of predicting aggression. Recent studies indicated that the most important predictor of physical violence, including while in a psychiatric institution, was a history of aggressive behavior in combination with male gender [6, 30, 31]. In our sample, the majority of aggressive events were perpetrated by male patients, compulsorily admitted with a psychotic disorder; besides these three factors, we could not find any relation for aggressive events in our sample. Controversially several studies found no gender differences regarding the exhibition of aggression among acute psychiatric patients [32–34].

In our sample, the time elapsed from admission to the aggressive event was 21.7 (27.7) days. Therefore, we consider that factors related to the length of stay could have triggered the aggressive event, therefore in our analysis we controlled for triggers (SOAS-R Item 1); time to event and length of stay.

In our sample, for patients with a “loose binding” antipsychotic medication, aggression was mainly directed at staff members. In contrast, for those with a “tight binding” antipsychotic medication, the victim of aggression was largely another patient. We intended to interpret these striking differences in the target of aggression with dynamic aspects. However, the triggers and characteristics of the aggressive events were somewhat similar; we were also unable to find interaction effects related to the targets of aggression. Studies investigating the relationship between the antipsychotic affinity for dopamine D2 receptors and patients**’** subjective well-being also had no conclusive findings [27]. Patient’s insight into need for medication has shown to be a protective factor for violent behavior in patients with schizophrenia [35]. The impact of adverse events and particularly akathisia on well-being is largely unexplored. Considering that akathisia is related to disruptive behavior and that patients spend most of the time with other patients (rather than with the staff), the opportunities that another patient becomes the target of aggression are strikingly higher. However, if this was the sole explanation of the results, we could expect more patients with “tight binding” antipsychotics as perpetrators, which is not the case.

In our sample, the most prescribed drugs were olanzapine and risperidone/paliperidone, overlapping with the current prescription praxis. The most commonly used second-generation antipsychotics were risperidone, olanzapine, clozapine, aripiprazole, and quetiapine [36, 37]. In contrast to previous findings were Long-Acting Injectables were prescribed as “loose binding” in 14.6% [38], Long-Acting Injectables were exclusively prescribed as “tight binding” antipsychotics here. We must consider that there might be different treatment preferences by physicians and nurses.

We limited our sample to patients with a psychotic disorder because the indication for antipsychotic medication is clearly stated for these patients (independent of disruptive, violent, or aggressive behavior). Since our analysis focused on the relationship of antipsychotics with aggressive events, these patients were required to take the prescribed antipsychotic medication. To further delimit the effects of antipsychotics, we included other prescribed psychoactive medications, such as mood stabilizers and anxiolytics, in the analysis. This approach led us to reduce the bias potentially derived from medication other than antipsychotics.

### Limitations

This study is subject to several limitations. First, the modest size of our sample limits the generalizability of our results. Still, the sample only contains severe aggression events, which are by definition rare. Only legally liable events that have been registered in our clinical incident system could be analyzed. Second, due to the small sample size and low numbers of prescribed antipsychotics, we could not analyze single antipsychotic drugs or attribute a causality to the correlation found. The use of non-parametric testing was intended to overcome the limitations derived from the low sample size and increase the validity of the results. We used the general linear model to analyze the influence and interaction of the different factors. In the study period of four years, we registered 61 incidents for 17,901 (0.34%) of all consecutive admissions; and 51 events in 4,394 (1.16%) perpetrated by patients with a psychotic disorder. Although desirable, the inclusion of a larger collection period was not possible since, for the time before January 2018, the collection of aggressive events was not systematically conducted. For a refined analysis, we also miss information that might play a role in aggressive behavior, like the kind and rate of adverse events or cognitive deficits. Third, following prior research [3] we included both psychotic patients with schizophrenia spectrum disorder and bipolar disorder with psychotic symptoms thus the results have to be interpreted as transdiagnostic correlate of psychotic symptoms and we cannot relate to individual diagnoses due to the small sample size of diagnostic subgroups. Fourth, even though history of violence is an important predictor of future aggressive behavior[3], we do not know the number and characteristics of severe aggressive events occurring before hospitalization (i.e., leading to hospitalization) as this information could not be collected due to lack of standardized reporting of prior aggressive behavior. We also do not know the frequency of less severe aggressive events (e.g., verbal assault) and the history of mechanical restraint/seclusion.

## Conclusions

In conclusion, our analysis uncovered a robust correlation between the antipsychotic medication prescribed and the focus of aggression in patients with a psychotic disorder. This result, especially its markedness, is astonishing and has potential clinical implications. Since our study is explorative, our results show a correlation, and we cannot derive causality from them. Therefore, we can merely speculate on the reasons for our findings. Nevertheless, the findings underscore the significance of vigilant clinical monitoring, particularly in regard to infrequent events. While our study did not provide definite answers, it paves the way for further inquiries and more refined questions.

The study raises up the question if individual antipsychotic agents should be preferred with a view to cases where reducing aggressive behavior seems to be a primary aim of therapy. Therefore, a prospective randomized study comparing the use of different substances with regard to the occurrence of aggressive events is needed.

## Data Availability

The datasets analyzed during the current study are available from the corresponding author on reasonable request due to ethical limitations.

## Abbreviations

CPE: Chlorpromazine Equivalents
DDD: Daily Defined Dosee
HoNOS: Health of the Nation Outcome Scale
ICD-10: International Statistical Classification of Diseases and Related Health Problems, 10th revision
LB: Loose binding
SD: Standard Deviation
SOAS-R: Staff Observation Aggression Scale - revised version
TB: Tight binding
